# Detection and Characterization of a Biochemical Signature Associated with Diabetic Nephropathy Using Near-infrared Spectroscopy on Tissue Sections

**DOI:** 10.3390/jcm8071022

**Published:** 2019-07-12

**Authors:** Sander De Bruyne, Jo Van Dorpe, Jonas Himpe, Wim Van Biesen, Sigurd Delanghe, Marijn M. Speeckaert, Joris R. Delanghe

**Affiliations:** 1 Department of Clinical Chemistry, Microbiology and Immunology, Ghent University, 9000 Ghent, Belgium; 2 Department of Pathology, Ghent University Hospital, 9000 Ghent, Belgium; 3 Department of Internal Medicine, Nephrology Division, Ghent University Hospital, 9000 Ghent, Belgium; 4 Research Foundation Flanders, 1000 Brussels, Belgium

**Keywords:** near-infrared spectroscopy, diabetic nephropathy, post-translational modifications, renal tissue

## Abstract

Histological evaluation of renal biopsies is currently the gold standard for acquiring important diagnostic and prognostic information in diabetic nephropathy (DN) patients. Nevertheless, there is an unmet clinical need for new biomarkers that allow earlier diagnosis and risk stratification. As biochemical changes in tissues must precede any symptomatic or morphological expression of a disease, we explored the potential of near-infrared (NIR) spectroscopy in the detection of a biochemical signature associated with DN. Kidney tissue sections were investigated using NIR spectroscopy, followed by principal component analysis and soft independent modelling of class analogy. A biochemical signature indicative of DN was detected, which enabled perfect discrimination between tissue sections with normal histological findings (*n* = 27) and sections obtained from DN patients (*n* = 26). Some spectral changes related to carbamoylation and glycation reactions appeared to be similar to the ones obtained in patients with DN. In addition, treatment with the deglycating enzyme fructosamine-3-kinase resulted in partial to pronounced restorations of the spectral pattern. Significant relationships were found between spectral features and laboratory parameters indicative of glycemic and uremic load, such as hemoglobin A1c, urea, creatinine, estimated glomerular filtration rate, and proteinuria. The presented method could be a useful tool to complement histopathological analysis in order to prevent or delay further disease progression, especially in the setting of post-transplant surveillance kidney biopsies.

## 1. Introduction

Around 20%–40% of patients with type 1 or type 2 diabetes mellitus (DM) develop diabetic nephropathy (DN) [1]. The latter is a clinical syndrome characterized by persistent albuminuria (>300 mg/24 h or > 300 mg/g creatinine), a decline in glomerular filtration rate (GFR), elevated blood pressure, and an excess cardiovascular morbidity and mortality [1,2]. It is the single most common cause of end-stage renal disease (ESRD) in many parts of the world, including Europe and the United States [1,3]. While kidney transplantation (Tx) is often an effective treatment for ESRD, transplant failure is common [4].

Monitoring of kidney transplants by obtaining serial serum creatinine levels is a widely accepted part of post-Tx management. However, due to problems with sensitivity and specificity, some programs supplement serial serum creatinine levels with surveillance kidney biopsies [5]. At the moment, histological evaluation of renal biopsies remains the gold standard for acquiring important diagnostic and prognostic information. Unfortunately, early evidence of DN, such as glomerular basement membrane thickening and mesangial expansion, is often not seen until several years after Tx [4,6]. Consequently, there is an unmet clinical need for new biomarkers that allow earlier diagnosis and risk stratification [7]. The early detection of recurrent DN could allow for more focused treatment in order to delay its progression [1]. As changes in the biochemical composition and structure must precede any symptomatic or morphological expression of a disease, vibrational spectroscopy is an ideal candidate for the early detection of biochemical signatures associated with the generation and progression of disease [8].

Detection of DN using vibrational spectroscopy was first reported by Varma et al. [4], who employed spectroscopic imaging in the mid-infrared (MIR) range to identify early biochemical changes associated with recurrence of disease in transplant patients prior to histologic changes. In the complex situation of DN, chronic kidney disease and DM accelerate protein molecular ageing through an increased intensity of non-enzymatic post-translational modifications (NEPTMs), such as glycation and carbamoylation [9,10]. In addition, glycation of tissue proteins has been associated with development of DN [11]. Near-infrared (NIR) spectroscopy is a different spectroscopic technique well-suited to detect post-translational modifications in a simple and non-destructive way. In this paper, we explored for the first time the potential of NIR spectroscopy to identify and unravel a biochemical signature associated with DN on stained tissue sections.

## 2. Experimental Section

### 2.1. Study Population

The control group consisted of 27 renal cortex biopsy samples, with normal histological findings, obtained from 22 post-Tx patients with DM (median time post-Tx: 90 days, IQR: 90.0–120.0 days), 3 non-post-Tx patients with DM, and 2 non-post-Tx patients without DM. Donor kidneys were derived from patients without DM. The study population group included 26 patients with different stages of DN. Apart from patients with DN, also 11 patients with DM and another renal pathology (renal cell carcinoma (RCC), 10 clear cell and 1 papillary) but no DN were investigated to explore the discriminative power of the technique. Diagnosis was based on the histological evaluation of renal biopsies by trained pathologists. Characteristics of the study population are summarized in Table 1. The study was approved by the local ethics committee (Belgian registration number B670201734663). The authors complied with the World Medical Association Declaration of Helsinki regarding ethical conduct in research involving human subjects.

### 2.2. Determination of Routine Laboratory Parameters

Hemoglobin A1c (HbA1c) was analyzed on ethylenediaminetetraacetic acid (EDTA) blood samples using a Menarini 8160 high-performance liquid chromatography system (Menarini, Firenze, Italy). Subjects were characterized as having diabetes based on the American Diabetes Association criteria [12]. Serum creatinine (mg/dL) and urea (mg/dL) were determined using a compensated rate-blanked picrate assay and an enzymatic method on a Cobas 8000 platform (Roche, Mannheim, Germany), respectively. The estimated glomerular filtration rate (eGFR) was calculated using the Chronic Kidney Disease Epidemiology Collaboration (CKD-EPI) 2009 formula [13]. Total urinary protein (g/g creatinine) was assayed by a pyrogallol red-molybdate method on a Cobas 8000 system (Roche Diagnostics, IN, USA) [14].

### 2.3. Preparation of Tissue Sections

For the ex vivo model, renal cortex tissue obtained through biopsy (16-gauge Tru-Cut Needle) was fixed with 10% neutral-buffered formalin for 6–24 h. After fixation, kidney biopsies were routinely processed using a Tissue-Tek^®^ VIP^®^ (Sakura, Torrance, CA, USA), after which they were embedded in paraffin. Subsequently, 2 μm tissue sections were cut, stained with hematoxylin and eosin (HE), periodic acid Schiff, Jones’ methenamine silver and Masson trichrome, and cover-slipped. Tissue sections were subjected to standard histological examination. NIR spectroscopic analysis was performed on archived HE-stained sections. In the cases of the (de)glycation and carbamoylation experiments, frozen sections (5 μm) were prepared using a clinical cryostat (Leica CM1950, Leica biosystems, Nussloch, Germany) at a temperature of −15 °C and mounted on SuperFrost^®^ microscope slides (Thermo Scientific, Waltham, MA, USA).

### 2.4. Carbamoylation and (de)Glycation of Tissue Sections

Biopsy material from a histologically normal cortex, obtained from a nephrectomy sample originating from a non-diabetic 88-year-old male donor, was used for the preparation of frozen tissue sections. Baseline measurements were performed on untreated samples. Carbamoylation of renal tissue was achieved by covering frozen tissue sections with a potassium cyanate solution (100 mM in phosphate buffered saline (PBS), Sigma-Aldrich, St. Louis, MO, USA), while glycation was achieved by covering the sections with a glucose solution (50 mM in PBS, Sigma-Aldrich, St. Louis, MO, USA at 37 °C for 72 h. Deglycation was initiated using ATP-dependent FN3K (Fitzgerald Industries International, Acton, MA, USA). A solution containing 0.016 g/L ATP-dependent FN3K in PBS was added (1:1) to a mixture of 5 mM ATP and 2 mM MgCl_2_ (Sigma-Aldrich) in PBS. Subsequently, glycated tissue sections were covered with the FN3K solution at 37 °C for 48 h. After incubation, tissue sections were carefully washed with distilled water and dried at 37 °C for 12 h. All experiments were performed independently on seven separate tissue sections. In addition, NIR spectra of L-lysine powder (≥98%, Sigma-Aldrich, St. Louis, MO, USA and its carbamoylated analogue, i.e., L-homocitrulline (≥95%, Santa Cruz Biotechnology, Dallas, TX, USA) were recorded.

### 2.5. Near-Infrared Spectroscopic Analysis

**Figure 1:**
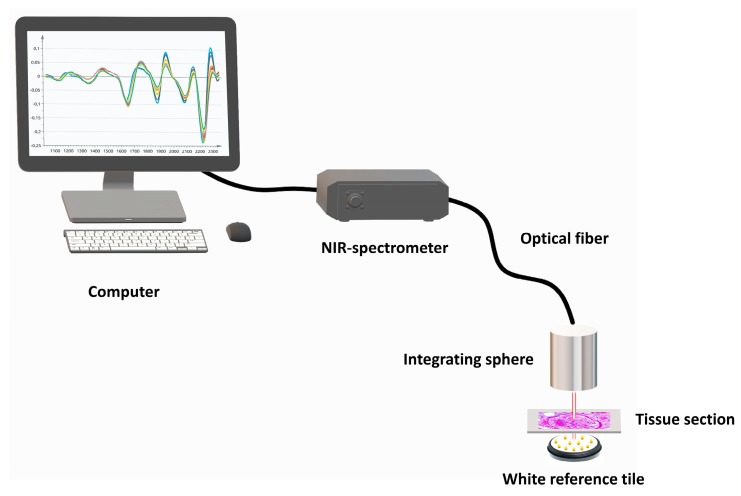
Schematic representation of the near-infrared (NIR) spectroscopy-based setup. The basic components include a computer, NIR-spectrometer, optical fiber, integrating sphere, and a white reference tile layered with immersion oil.

Spectral data were obtained at ambient temperature using a NIR spectrometer (AvaSpecNIR256-2.5-HSC, Avantes, Apeldoorn, The Netherlands), equipped with extended indium gallium arsenide (InGaAs) array technology. Tissue sections were placed onto a white reference tile, layered with immersion oil to eliminate loss of resolution due to different refractive surfaces (glass versus air). Subsequently, an immobilized 50 mm integrating sphere (AvaSphere-50-LS-HAL-6-S1, Avantes) with a 6 mm sample port diameter was placed directly onto the tissue sections for analysis. Analysis was performed in the renal cortex zone, indicated by a pathologist. Figure 1 illustrates the basic components of the proposed method. Spectra of the reflected light were recorded across the range of 1038–2354 nm at a resolution of 13 nm (128 averaged scans). All samples were analyzed in batch to minimize external variabilities.

### 2.6. Multivariate Data Analysis

NIR spectral data analysis was performed using SIMCA^®^ software version 15.0 (MKS Data Analytics Solutions, Umeå, Sweden). To remove irrelevant light scatter and standardize the spectroscopic signal, different preprocessing methods, such as standard normal variate (SNV), derivatives, and Savitzky-Golay (SG) smoothing, were examined. SNV eliminates additive baseline offset variations and multiplicative scaling effects, which may be induced by differences in sample density and sample-to-sample measurement variations. For more complex spectra (e.g., biological spectra), differentiation is utilized to accentuate small structural differences and reduce baseline effects, which facilitates spectral discrimination [15,16]. Furthermore, derivatives enhance spectral resolution, which is helpful in resolving and locating overlapping bands. In the first derivative, resolution is enhanced since the rate of change of absorbance (A) with respect to wavelength (λ) is examined (dA/dλ). The second derivative measures alterations in the rate of change of absorbance (d^2^A/dλ^2^) [15]. SG smoothing can be performed to reduce the level of noise, while keeping the spectral details [16].

After preprocessing, spectral data were analyzed by unsupervised pattern recognition methods, such as principal component analysis (PCA), and supervised pattern recognition methods, such as soft independent modelling of class analogy (SIMCA). PCA is a multivariate projection method that is used to analyze the interrelationships among a large number of spectral data points and to explain these variables into a few orthogonal principal components (PCs), such that an overview of the data is obtained with a minimum loss of information [15]. The first PC is the line in the K-dimensional space that best approximates the data in the least squares sense and passes through the average point. One PC is often insufficient to model the systematic variation of a data set, and therefore a second PC is calculated. The second PC is orthogonal to the first PC, goes through the average point, and ameliorates the approximation of the X-data as much as possible. Two PCs together form a plane, and by projecting all the observations onto this subspace and plotting the results, the structure of the investigated data set can be visualized [15,17]. Scores are defined as the coordinates of the observations on this plane, while a score plot represents the plotting of such a projected configuration. However, more PCs are often required to adequately summarize the information in a data set [15,17].

Soft independent modelling of class analogy (SIMCA) was used to evaluate the predictive properties of NIR spectroscopy on tissue sections. This method models each class of samples separately by executing a PCA on each class and defines the optimal number of PCs required to describe each class of samples individually by using a cross-validation procedure [15]. During cross-validation, observations are omitted, predicted, and compared to the actual values using different numbers of PCs. The same procedure is repeated until every data point has been kept out once. The PC model that produces the minimum prediction error is retained. In contrast to most standard discrimination techniques, SIMCA can work with as few as 10 samples per class without problems arising from collinearity and chance classification.

### 2.7. Statistical Analysis

Statistical analysis was carried out using MedCalc Version 18.11 (MedCalc Software, Mariakerke, Belgium). Differences between patient groups were assessed using the Mann-Whitney U test. To investigate correlations between non-normal continuous variables, Spearman’s rho (ρ) was calculated. A *p*-value < 0.05 was considered a priori to be statistically significant.

## 3. Results

### 3.1. Exploration of Discriminative Spectral Features in Diabetic Nephropathy Patients

**Figure 2:**
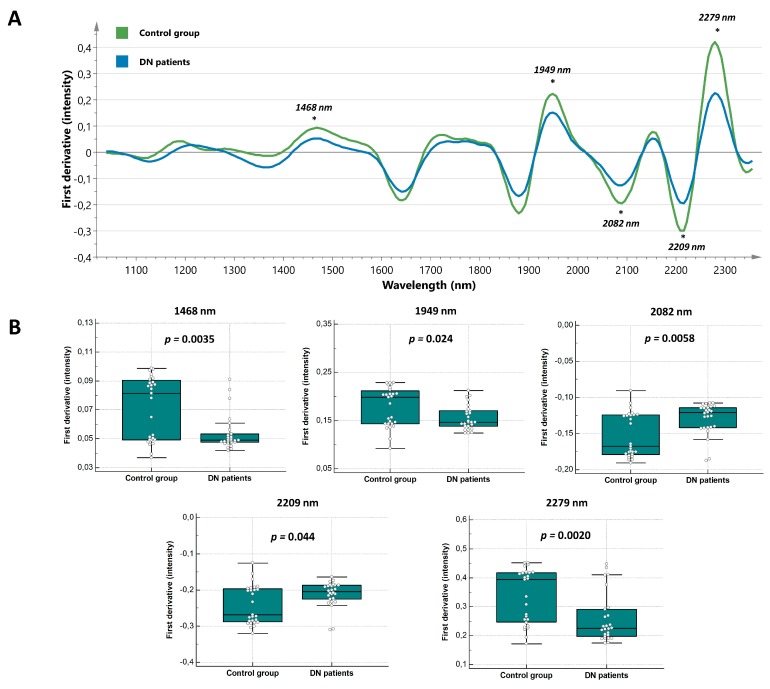
Discriminative spectral features in diabetic nephropathy (DN) patients. (**A**) First derivative of the median spectra from the control group (green line, *n* = 27) and DN patients (blue line, *n* = 26). Statistically significant differences (Mann-Whitney U test) in peak intensities between both groups are indicated with an asterisk. In first derivative, resolution is enhanced since the rate of change of absorbance (**A**) with respect to wavelength (λ) is examined (dA/dλ). (**B**) Box and whisker plots showing decreased intensities at 1468 nm, 1949 nm, and 2279 nm in the group of DN patients, compared to the controls. In contrast, the intensities at 2082 nm and 2209 nm showed significant increases.

Initially, potential zones of interests were identified by comparison of the SNV normalized, first derivative, and SG smoothed (15 points) median spectra of the control and DN group (Figure 2A). Since spectral changes were noted over the entire spectral range, the statistical significance of each region was tested by applying a Mann-Whitney U test on its peak intensity. The peak intensities at 1468 nm, 1949 nm, and 2279 nm were significantly lower (*p* = 0.0035, *p* = 0.024, and *p* = 0.0020, respectively), while the intensities at 2082 nm and 2209 nm were significantly higher (*p* = 0.0058 and *p* = 0.044, respectively) in patients with DN in comparison with the control subjects (Figure 2B). Next to these isolated peak intensities, statistically significant differences were found in the following regions: 1189–1208 nm, 1273–1293 nm, 1384–1552 nm, 1603–1655 nm, 1719–1738 nm, 1910–1968 nm, 2025–2120 nm, 2184–2209 nm, 2241–2316 nm, and 2329–2354 nm. With relevant wavelengths identified, we proceeded to evaluate the potential of NIR spectroscopy to detect DN by applying SIMCA. To maximize the predictive potential, several classification models based on narrower spectral ranges were developed. Eventually, a classification model based on the spectral region ranging from 1700–2165 nm was able to classify all control and DN samples with perfect accuracy.

During classification, the probability of belonging to a specific class is calculated for each sample in the dataset. Samples with a probability of > 0.10 are considered to be inside the 90% confidence interval (CI) of the normal probability curve, values between 0.10 and 0.05 are considered to be inside 95% CI, and samples with a probability < 0.05 are deemed to be outside the class (outside 95% CI). However, a model was applied that assigned each observation only to the nearest class. It is interesting to note that 4 control patients, who were correctly classified to the control group, also showed a probability > 0.05 for the class model of DN patients, and consequently, were also deemed to be inside the 95% or 90% CI of the DN class model. One of these patients, a 15-year old girl with DM who initially underwent a biopsy with normal histological findings in the context of proteinuria (the sample used for NIR analysis), eventually was diagnosed with DN on a biopsy six years later. The other 3 patients did not undergo surveillance biopsies after the first biopsy. Nevertheless, one of these patients, a 45-year old male with DM who underwent a biopsy 3 months after Tx with normal histological findings, developed a persistent and progressive albuminuria (>300 mg/g creatinine) with declined eGFR in the follow-up period. Furthermore, a 55-year old women with DM, who also underwent a biopsy 3 months after Tx with normal histological findings, developed a longstanding proteinuria 3 years after biopsy. Taken together, these findings could suggest a potential role of NIR spectroscopy in the very early detection of DN prior to histological abnormalities. No follow-up data were available for the third post-Tx control patient, since a nephrectomy of the donor kidney was performed shortly after biopsy due to arterial thrombosis. 

### 3.2. Unravelling The Biochemical Nature of Discriminative Spectral Features

**Figure 3:**
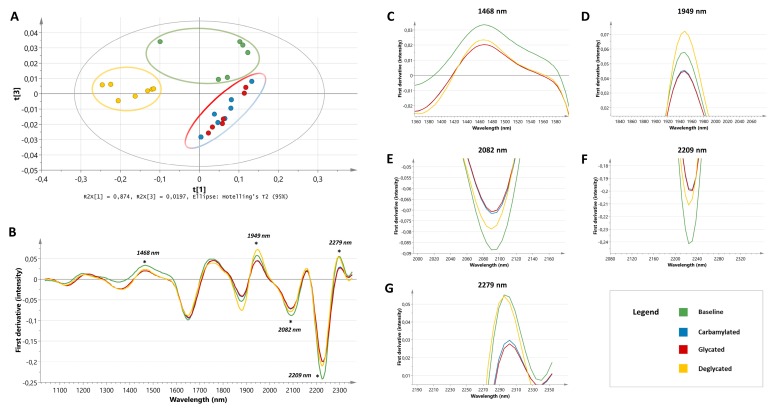
Spectral signature of (de)glycated and carbamoylated tissue sections. (**A**) Score plot showing clear clustering of three groups: baseline samples (green dots), deglycated samples (yellow dots), and a separate group with the glycated and carbamoylated samples (red and blue dots, respectively) based on the 1700–2165 nm spectral range. (**B**) First derivative of the mean spectra (full spectral range) from the baseline samples (green line), carbamoylated (blue line), glycated (red line), and deglycated samples (yellow line). Close-up on the zones of interest at 1468 nm (**C**), 1949 nm (**D**), 2082 nm (**E**), 2209 nm (**F**), and 2279 nm (**G**).

To unravel the biochemical nature of the most striking spectral differences in DN patients, (de)glycation and carbamoylation experiments were performed on unstained tissue sections (Figure 3). The same preprocessing steps as applied in the ex vivo model were executed and PCA was performed on the most discriminative spectral region previously found in the spectra of DN patients (i.e., 1700–2165 nm). The PCA model identified two outliers that were placed far outside the 95% confidence interval of the Hotelling’s plot: one baseline sample and one glycated tissue section. Since outliers might seriously bias the mean spectra, both samples were excluded. The resulting score plot showed clear clustering of three groups (Figure 3A): baseline samples (green dots), deglycated samples (yellow dots), and a separate group with the glycated and carbamoylated samples (red and blue dots, respectively). With regard to the latter, spectral changes observed after glycation and carbamoylation seemed to be very similar.

**Figure 4:**
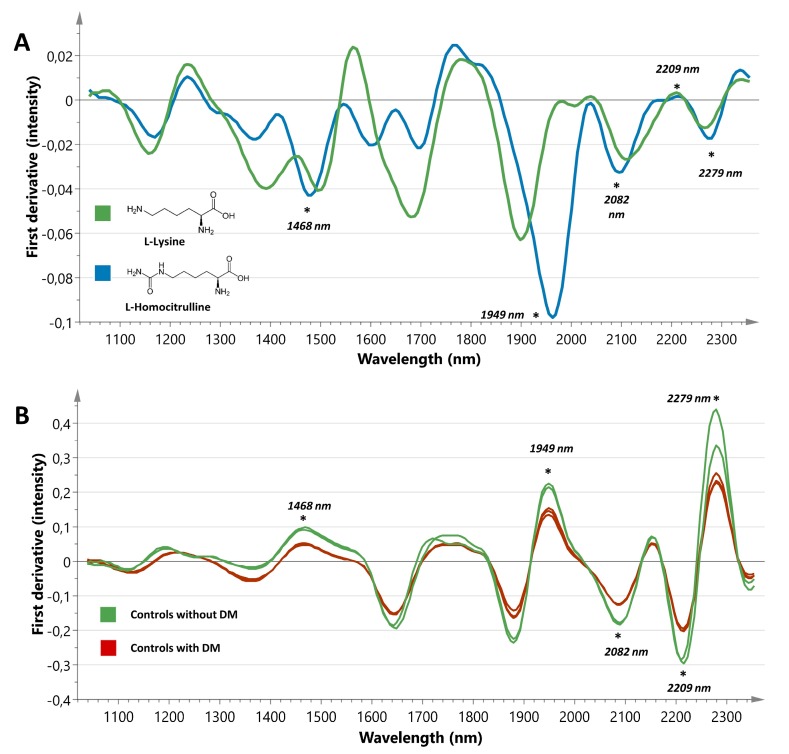
(**A**) First derivative spectra of lysine (green line) and homocitrulline powder (blue line). The peaks of interest are indicated with an asterisk. (**B**) First derivative spectra of non-post-transplant patients without DM (*n* = 2) and non-post-transplant patients with DM (*n* = 3).

Mean spectra of baseline, (de)glycated, and carbamoylated tissue sections were visually compared to expose differences (Figure 3B). The peak at 1468 nm, mainly attributed to N-H combination bands from CONH_2_ groups [18], showed a decrease in intensity after the carbamoylation process (Figure 3C). In addition, the first derivative spectrum of homocitrulline showed a modest decrease in the region intensity compared to lysine (Figure 4A). Furthermore, it is known that O-H (2*v*) groups can provoke spectral influence around this region [18]. After glycation, a similar decrease was observed in the intensity of the 1468 nm peak. Deglycation with FN3K had little effect on the peak intensity. The peak located at 1949 nm became less expressed after carbamoylation and glycation (Figure 3D). This can be explained by the fact that this region can be assigned to N-H combination bands from CONH_2_ groups, as well as O-H stretching and HOH bending combinations [18]. These findings were confirmed by the spectrum of homocitrulline, showing a strong decrease in the 1949 nm peak intensity compared to lysine (Figure 4A). Furthermore, FN3K treatment provoked a complete restoration, even beyond baseline level, of the glycated tissue sections. Since a similar decrease was noted in the spectra of DN patients, it is likely that carbamoylation and glycation are significant contributors to this spectral finding. Furthermore, the 2082 nm peak, which is also associated with CONH_2_ groups and O-H bending and C-O stretching combinations [18], showed an increased intensity after both the carbamoylation and glycation process. The latter was confirmed by a partial restoration of the increased peak intensity in the glycated tissue sections using FN3K (Figure 3E). However, the spectra of lysine and homocitrulline did not reveal spectral differences, in line with our carbamoylation findings (Figure 4A). The 2209 nm peak, associated with C-H stretching and C = O combination bands [18], showed a marked intensity increase after glycation and carbamoylation. FN3K treatment caused a modest restoration of the increased intensity observed in the glycated samples (Figure 3F). No clear spectral changes were observed in the spectra of lysine and homocitrulline (Figure 4A). At last, the peak intensity at 2279 nm, associated with CONH_2_ groups and O-H, C-O stretching combinations [18], showed a minimal decrease after carbamoylation and glycation (Figure 3G). While a rather subtle intensity decrease was found in the spectrum of homocitrulline compared to lysine (Figure 4A), FN3K treatment resulted in a complete spectral restoration of the glycated samples towards baseline level.

### 3.3. Discriminative Power of The Biochemical Signature

Figure 4B shows the spectra of two non-post-Tx control patients without DM and three non-post-Tx control patients with DM. Comparison of spectra revealed striking similarities with the changes observed during the (de)glycation experiment in the full spectral range. Although carbamoylation and glycation-induced spectral changes in some regions also appeared to be similar to the ones obtained in DN patients, it is likely that these NEPTMs can only be partially associated with the biochemical signature observed in DN patients. This statement can be strengthened by the fact that all control patients with DM but without DN were correctly classified as controls in the classification model mentioned above. Furthermore, one patient with DM from the control group, who underwent a biopsy 10 years after Tx, was correctly classified in the control group despite having a longstanding high glycemic and uremic load during follow-up.

In addition, tissue sections of patients with DM and another renal pathology (RCC) were analyzed to investigate whether the biochemical signature is discriminatory for presence of true DN in patients with DM. A classification model based on the first derivative of the whole spectral range (SNV normalized and SG smoothed) was designed to discriminate controls, DN patients, and patients with DM and RCC. The model generated a correct classification ratio of 93.8%. In the control group (*n =* 27), one sample was misclassified as DN, while 2 samples were incorrectly classified as DM with RCC. In the DN group (*n =* 26), one sample was misclassified as DM with RCC. At last, all tissue sections in the group of DM patients with RCC (*n =* 11) were correctly classified. Consequently, we can assume that the biochemical signature seems to be discriminatory for presence of true DN in patients with DM, and is more than just a reflection of a patient’s glycation or carbamoylation status.

### 3.4. Correlation of Spectral Markers with Routine Laboratory Parameters and Age

**Figure 5:**
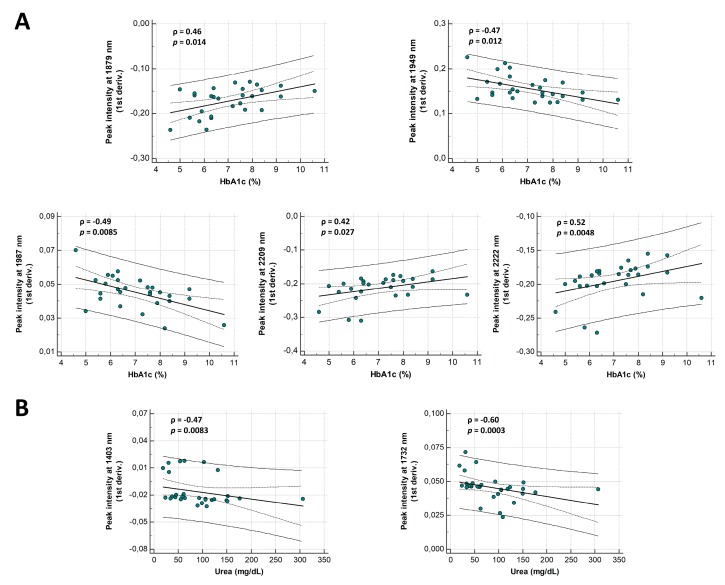
Correlation of spectral markers with routine laboratory parameters. (**A**) Scatterplots illustrating Spearman’s correlations between first derivative intensities at 1879 nm, 1949 nm, 2209 nm, 1987 nm, 2222 nm, and hemoglobin A1c (HbA1c) (%). (**B**) Scatterplots illustrating correlations between first derivative intensities at 1403 nm, 1732 nm, and urea (mg/dL). The solid and dashed lines represent the 95% prediction interval and 95% confidence interval, respectively.

Correlations of spectral markers with urea, creatinine, eGFR, HbA1c, proteinuria, and age were investigated. Tissue sections obtained from post-Tx patients (*n* = 22) were excluded since the detected NEPTMs can reflect the past dynamics of average blood urea and glucose levels from the donor and not from the acceptor. HbA1c values were unavailable in three DN patients. A significant correlation with HbA1c was found for the peak intensities at 1949 nm (ρ = −0.47, *p* = 0.012, Figure 5A) and 2209 nm (ρ = 0.42, *p* = 0.027, Figure 5A), which is in-line with the results obtained during the (de)glycation experiment. Furthermore, a significant correlation with proteinuria was noted for the peak at 1468 nm (ρ = −0.39, *p* = 0.039) and 1949 nm (ρ = −0.39, *p* = 0.038). While the 2279 nm peak showed a nearly significant correlation with proteinuria (ρ = −0.36, *p* = 0.053), no significant correlations were observed for the other peaks of interest.

Nonetheless, next to the initially defined peaks of interest, several other spectral features showed significant correlations with HbA1c and urea. The peak intensity at 1879 nm, 1987 nm, and 2222 nm showed the best correlations with HbA1c (ρ = 0.46, *p* = 0.014; ρ = −0.49, *p* = 0.0085; and ρ = 0.52, *p* = 0.0048, respectively, Figure 5A). The link of these regions with the glycation status was confirmed by the glycation experiment, revealing spectral changes in all these regions and FN3K treatment causing marked spectral restorations. Moreover, best correlations with urea were found for the intensities at 1403 nm and 1732 nm (ρ = −0.47, *p* = 0.0083; ρ = −0.60, *p* = 0.0003, respectively; Figure 5B). The region around 1403 nm can be assigned to N-H (2*v*) symmetric vibrations, while the intensity at 1732 nm can be linked to CONH_2_ groups (specifically due to C = O hydrogen bonded to the N-H of the peptide link termed the α-helix structure) [18]. Both regions became less expressed after carbamoylation and the spectrum of homocitrulline showed subtle intensity decreases in these regions compared to lysine (Figure 4A). In addition, significant correlations with creatinine and eGFR were found for both the intensity at 1403 nm (ρ = −0.44, *p* = 0.012; ρ = 0.47, *p* = 0.0072, respectively) and 1732 nm (ρ = −0.47, *p* = 0.0084; ρ = 0.47, *p* = 0.0070, respectively). No significant correlations were found with age. 

## 4. Discussion

In the present paper, we have demonstrated for the first time the use of NIR spectroscopy to assess DN in renal biopsies from a single stained tissue section. The spectral range between 1700–2165 nm, a region potentially and partially associated with both carbamoylation (CONH_2_ groups) and glycation (O-H stretching and HOH bending combinations, O-H bending, and C-O stretching combinations) reactions, allowed perfect discrimination. In addition, several control patients, who were also deemed to be inside the 95% or 90% CI of the DN class model, developed histological or clinical signs associated with DN during follow-up. This could suggest a potential role of NIR spectroscopy in the very early detection of DN prior to histological abnormalities. Carbamoylation and glycation derived spectral changes in some regions appeared to be similar to the ones obtained in DN patients. Based on our results, NEPTMs are likely to be contributors to some of the observed spectral changes. This is further supported by the significant relationships found between spectral features and routine laboratory parameters, such as HbA1c, urea, creatinine, eGFR, and proteinuria. When isocyanic acid (carbamoylation) or glucose (glycation) makes a stable attachment to a protein, it takes on the half-life of that protein [19]. As kidney proteins are characterized by a long half-life, the spectral features can be seen as time-integrated markers, which allow to reveal the past dynamics of average blood urea and glucose levels within a long-time window. Nevertheless, since we were able to discriminate patients with DM but without DN from patients with DN, we can assume that the biochemical signature is not solely a reflection of a patient’s glycation or carbamoylation status. This is of importance, as not all patients with kidney disease and diabetes do have DN [20].

Our results are in line with the MIR imaging results reported by Varma et al. [4], wherein a biochemical signature of DN was linked to tissue glycation [21]. However, it has to be mentioned that their study was faced with some limiting factors such as the inclusion of only a few study participants, the lack of an in vitro model to better characterize the relationship between spectral findings and their biochemical nature, and more importantly the use of a complex and expensive analytical technique, which makes the method only suitable for clinical research purposes. Therefore, we developed a practical and portable method based on a considerably higher amount of study participants in an entirely different range of the electromagnetic spectrum by using a different analytical technique. Our novel method is not restricted to very specialized laboratories and can be performed on routine stained tissue sections without the need for additional sample preparation. However, in contrast to imaging, our setup is not able to specifically target key structures in renal biopsies, but provides a rather general insight into tissue biochemistry.

Next to the analytical innovation, the proposed method has the ability to impact clinical care on multiple fronts. Treatment for DN is often not very satisfying, possibly because renal involvement is discovered too late using the conventional diagnostic techniques [4]. Since biochemical alterations precede histopathological changes, early NIR identification of DN could be useful to complement histopathological analysis in order to prevent or delay further disease progression with more focused interventions, especially in the setting of post-Tx surveillance kidney biopsies. In addition, NIR spectroscopy may also be helpful in monitoring the effect of instituted therapies. Since there is a need for better biomarkers that can predict which patients with DM are at highest risk for progression of their disease [22], future research could focus on the potential of the spectral features as prognostic biomarkers for progressive DN. Nevertheless, studies on a larger number of patients are needed to confirm the external validity of our results. Full validation studies with adequate training and test sets have to be designed.

In conclusion, a biochemical signature associated with DN was detected in an objective and non-destructive way by NIR spectroscopic analysis of stained tissue sections without additional sample preparation. The reported method could be a useful adjunct to standard histopathological analysis. However, large-scale prospective follow-up studies are indispensable to further assess its clinical value in the early detection of DN prior to the occurrence of histological abnormalities.

## Figures and Tables

**Table 1:** Characteristics of the study population.

Variable	Ctr (*n =* 27)	DN (*n =* 26)	DM and RCC (*n =* 11)	Ctr vs. DN *p*-Value	Ctr vs. DM and RCC *p*-Value	DN vs. DM and RCC *p*-Value
Age (yr)	55 (47–61)	60 (50–66)	70 (66–73)	N.S.	<0.0001	0.0061
Women (%)	70	77	91	N.S.	N.S.	N.S.
DM (%)	89	100	100	N.S.	N.S.	N.S.
Creatinine (mg/dL)	1.2 (1.0–1.9)	1.9 (1.3–3.3)	1.4 (1.3–1.5)	0.0096	N.S.	N.S.
eGFR (mL/min/1.73 m^2^)	54 (38–79)	36 (16–50)	53 (40.0–54)	0.0091	N.S.	N.S.
Urea (mg/dL)	44.0 (31.8–66.3)	96.0 (54.0–124.0)	38.0 (33.0–41.0)	0.0011	N.S.	0.0011
Proteinuria (g/g creat)	0.2 (0.03–0.4)	2.7 (2.3–6.9)	0.0 (0.0–0.12)	<0.0001	N.S.	<0.0001
HbA1c (%)	6.6 (6.0–7.4)	7.4 (6.2–8.1)	6.6 (6.0–7.7)	N.S.	N.S.	N.S.

Data are presented as median (interquartile range). Note: *p*-value = Mann-Whitney U test. Abbreviations: ctr = control; create = creatinine; DM = diabetes mellitus; DN = diabetic nephropathy; eGFR = estimated glomerular filtration rate; HbA1c = hemoglobin A1c; N.S. = non-significant; RCC = renal cell carcinoma; yr = year.

## References

[1] Persson F., Rossing P. (2018). Diagnosis of diabetic kidney disease: State of the art and future perspective. Kidney Int. Suppl..

[2] Papadopoulou-Marketou N., Chrousos G.P., Kanaka-Gantenbein C. (2017). Diabetic nephropathy in type 1 diabetes: A review of early natural history, pathogenesis, and diagnosis. Diabetes Metab. Res. Rev..

[3] Tuttle K.R., Bakris G.L., Bilous R.W., Chiang J.L., De Boer I.H., Goldstein-Fuchs J., Hirsch I.B., Kalantar-Zadeh K., Narva A.S., Navaneethan S.D. (2014). Diabetic kidney disease: A report from an ADA consensus conference. Diabetes Care.

[4] Varma V.K., Kajdacsy-Balla A., Akkina S.K., Setty S., Walsh M.J. (2016). A label-free approach by infrared spectroscopic imaging for interrogating the biochemistry of diabetic nephropathy progression. Kidney Int..

[5] Josephson M.A. (2011). Monitoring and managing graft health in the kidney transplant recipient. Clin. J. Am. Soc. Nephrol..

[6] Adler S. (2004). Diabetic nephropathy: Linking histology, cell biology, and genetics. Kidney Int..

[7] Currie G., McKay G., Delles C. (2014). Biomarkers in diabetic nephropathy: Present and future. World J. Diabetes.

[8] De Bruyne S., Speeckaert M.M.M., Delanghe J.R. (2018). Applications of mid-infrared spectroscopy in the clinical laboratory setting. Crit. Rev. Clin. Lab. Sci..

[9] Nicolas C., Jaisson S., Gorisse L., Tessier F.J., Niquet-Léridon C., Jacolot P., Pietrement C., Gillery P. (2018). Carbamoylation is a competitor of glycation for protein modification in vivo. Diabetes Metab..

[10] Gorisse L., Pietrement C., Vuiblet V., Schmelzer C.E.H., Köhler M., Duca L., Debelle L., Fornès P., Jaisson S., Gillery P. (2016). Protein carbamoylation is a hallmark of aging. Proc. Natl. Acad. Sci. USA.

[11] Rodrigues L., Matafome P., Crisóstomo J., Santos-Silva D., Sena C., Pereira P., Seiça R. (2014). Advanced glycation end products and diabetic nephropathy: A comparative study using diabetic and normal rats with methylglyoxal-induced glycation. J. Physiol. Biochem..

[12] American Diabetes Association (2018). Classification and Diagnosis of Diabetes: Standards of Medical Care in Diabetes—2018. Diabetes Care.

[13] Levey A.S., Stevens L.A., Schmid C.H., Zhang Y.L., Iii A.F.C., Feldman H.I., Kusek J.W., Eggers P., Van Lente F., Greene T. (2014). A New Equation to Estimate Glomerular Filtration Rate. Ann. Intern. Med..

[14] Orsonneau J.L., Douet P., Massoubre C., Lustenberger P., Bernard S. (1989). An improved pyrogallol red-molybdate method for determining total urinary protein. Clin. Chem..

[15] De Bruyne S., Speeckaert R., Boelens J., Hayette M.P., Speeckaert M., Delanghe J. (2018). Infrared spectroscopy as a novel tool to diagnose onychomycosis. Br. J. Dermatol..

[16] Monteyne T., Coopman R., Kishabongo A.S., Himpe J., Lapauw B., Shadid S., Van Aken E.H., Berenson D., Speeckaert M.M., De Beer T. (2018). Analysis of protein glycation in human fingernail clippings with near-infrared (NIR) spectroscopy as an alternative technique for the diagnosis of diabetes mellitus. Clin. Chem. Lab. Med..

[17] Eriksson L., Johansson E., Kettaneh-Wold N., Trygg J., Wikström C., Wold S. (2006). Pca. Multi-and Megavariate Data Analysis Part 1, Basic Principles and Applications.

[18] Workman J., Weyer L. (2007). Practical Guide to Interpretive Near-Infrared Spectroscopy.

[19] Balion C.M., Draisey T.F., Thibert R.J. (1998). Carbamoylated hemoglobin and carbamoylated plasma protein in hemodialyzed patients. Kidney Int..

[20] Fiorentino M., Bolignano D., Tesar V., Pisano A., Van Biesen W. (2017). Renal biopsy in patients with diabetes: A pooled meta-analysis of 48 studies. Nephrol. Dial. Transplant..

[21] Delanghe J.R., Delanghe S.E., De Buyzere M.L., Speeckaert M.M. (2016). Infrared spectroscopic imaging for interrogating the carbohydrate biochemistry of diabetic nephropathy progression. Kidney Int..

[22] Brosius F.C., Pennathur S. (2013). How to find a prognostic biomarker for progressive diabetic nephropathy. Kidney Int..

